# Novelty application of multi-omics correlation in the discrimination of sulfur-fumigation and non-sulfur-fumigation Ophiopogonis Radix

**DOI:** 10.1038/s41598-017-10313-1

**Published:** 2017-08-30

**Authors:** Shengyun Dai, Zhanpeng Shang, Fei Wang, Yanfeng Cao, Xinyuan Shi, Zhaozhou Lin, Zhibin Wang, Ning Li, Jianqiu Lu, Yanjiang Qiao, Jiayu Zhang

**Affiliations:** 1 School of Chinese Pharmacy, Beijing University of Chinese Medicine, Beijing, 100102 China; 2 Beijing Hospital of Traditional Chinese Medicine, Capital Medical University, Beijing, 100010 China; 3 Beijing Research Institute of Chinese Medicine, Beijing University of Chinese Medicine, Beijing, 100029 China; 4 Shenzhen Research Institute, The Hong Kong University of Science and Technology, Shenzhen, 518057 China

## Abstract

A rapid and sensitive approach to differentiate sulfur-fumigated (SF) Ophiopogonis Radix based on Multi-Omics Correlation Analysis (MOCA) strategy was first established. It was characterized by multiple data-acquisition methods (NIR, HPLC, and UHPLC-HRMS) based metabonomics and multivariate statistical analysis methods. As a result, SF and non-sulfur fumigated (NSF) Ophiopogonis Radix samples were efficaciously discriminated. Moreover, based on the acquired HRMS data, 38 sulfur-containing discriminatory markers were eventually characterized, whose NIR absorption could be in close correlation with the discriminatory NIR wavebands (5000–5200 cm^−1^) screened by NIR metabonomics coupled with SiPLS and 2D-COS methods. This results were also validated from multiple perspectives, including metabonomics analysis based on the discriminatory markers and the simulation of SF ophiopogonin D and Ophiopogonis Radix sample. In conclusion, our results first revealed the intrinsic mechanism of discriminatory NIR wavebands by means of UHPLC-HRMS analysis. Meanwhile, the established MOCA strategy also provided a promising NIR based differential method for SF Ophiopogonis Radix, which could be exemplary for future researches on rapid discrimination of other SF Chinese herbal medicines.

## Introduction

Most Chinese herbal medicines (CHM) need to undergo post-harvest processing to convert raw materials into ready-to-use forms^1^. Over recent decades, sulfur-fumigation is becoming misused in processing some fresh harvested herbs, mainly because the sulfur dioxide (SO_2_) generated in the process can moisturize and bleach the medicinal herbs, retain freshness, and kill parasites^2^. Since the method was first applied into Dioscoreae Rhizoma in 1900, it has been widely applied in various CHM, such as Ophiopogonis Radix, Ginseng Radix *et* Rhizoma, Codonopsis Radix, Angelicae Sinensis Radix, *etc* ^1^. However, it has recently emerged as a controversial topic due to the potential detrimental effects to CHM, such as chemical transformation of inherent herbal constituents that consequently result in changing bioactivities, pharmacokinetics, and toxicities^3–6^. Thus, it is of great importance to develop a rapid and sensitive approach to ascertain the sulfur-fumigation state of a given medicinal herb for CHM quality control.

The rapid development of high-throughput technologies and computational frameworks of omics enables relative abundant and accurate data sets, which supplies tremendous help to monitor the system without intervening in its workings using tools that allow for global analysis of complex systems^7^. To best understand these emerged problems on sulfur-fumigation in CHM, an integrative approach with large-scale experiments (omics methodology based on different detection technologies), such as LC-MS, LC, and NIR, should be used as first-line^8–11^. Among them, NIR spectroscopy has been considered as an attractive and promising analytical method for pharmaceutical industry. In comparison with classical analytical methods, NIR analysis is rapid, non-destructive, and needs little or no sample preparation, which makes NIR more suitable for rapid CHM quality determination^12–15^. Moreover, it can provide both physical and chemical information about samples simultaneously^16–18^. However, the sensitivity and specificity of NIR cannot allow it to provide detailed composition of samples hence it cannot be used to reveal sulfur fumigation mechanism. Ultra-high-performance liquid chromatography (UHPLC) coupled with various detector arrays, such as diode array detector (DAD), evaporative light-scattering detector (ELSD), and electrochemical detector (ECD), *etc*, is the most frequently used analytical technology to perform discrimination with relative low cost and comprehensive detection applicability^19, 20^. Moreover, UHPLC coupled with tandem mass spectrometry (MS/MS) especially high-resolution (HR) MS/MS provides much higher detection speed, sensitivity, mass resolution, and accuracy. Therefore, it has already become a powerful approach for rapid identification of constituents in CHM and biological samples, which can be utilized to clarify underlying mechanisms^1, 21–25^. But other characteristics of LC-HRMS, such as high cost, complicated experimental operation, time-consuming, difficult to achieve *in-situ* testing, *ect*, severely hinder its application in the rapid separation and identification of CHM constituents. Based on above viewpoints, Multi-Omics Correlation Analysis (MOCA), covering spectroscopy, chromatography, and HRMS analysis, could be an efficient hyphenated technique to simultaneously achieve rapid CHM discrimination and inherent specific mechanism clarification.

**Figure 1:**
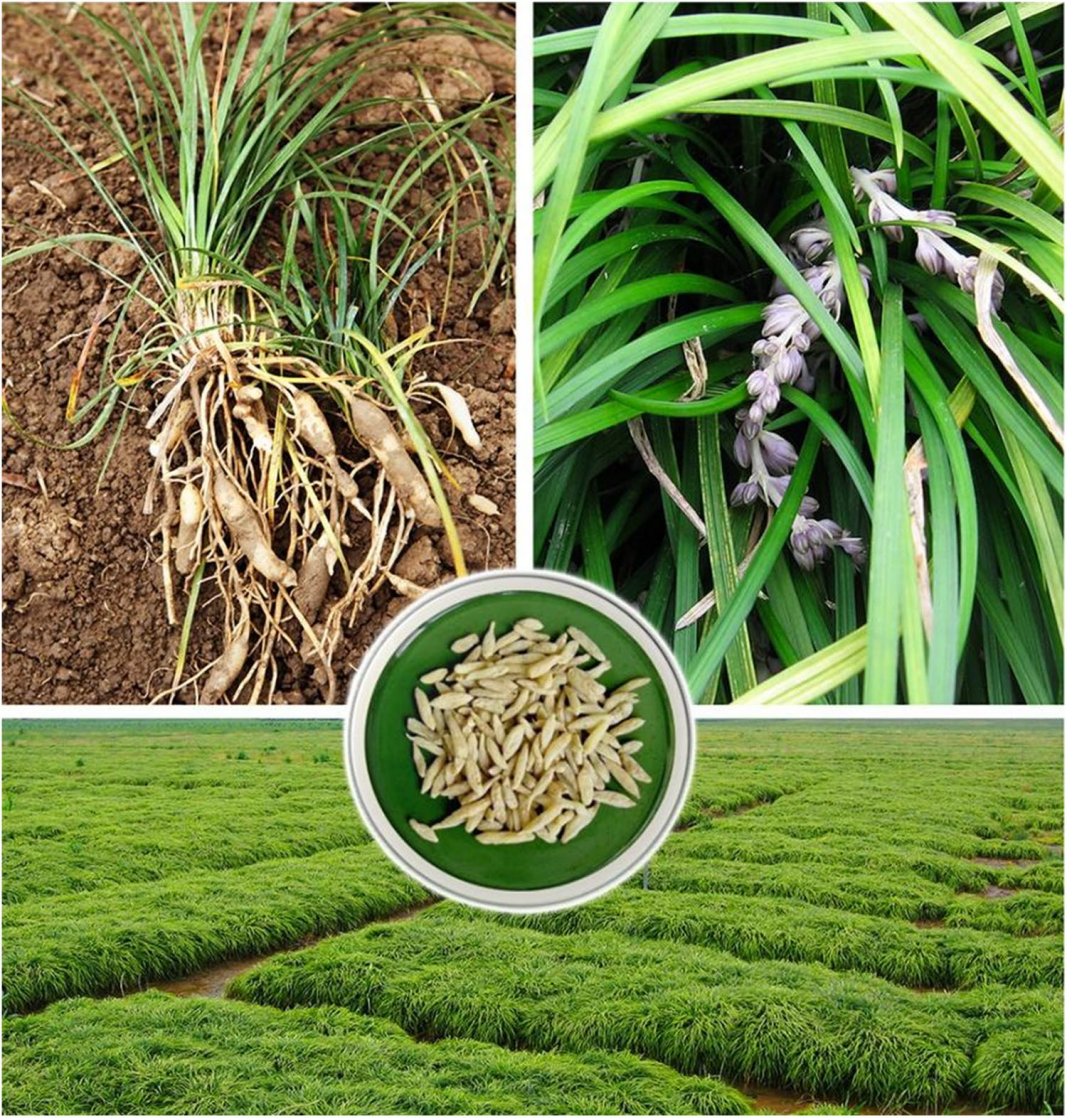
Plant characteristics of Ophiopogonis Radix.

**Figure 2:**
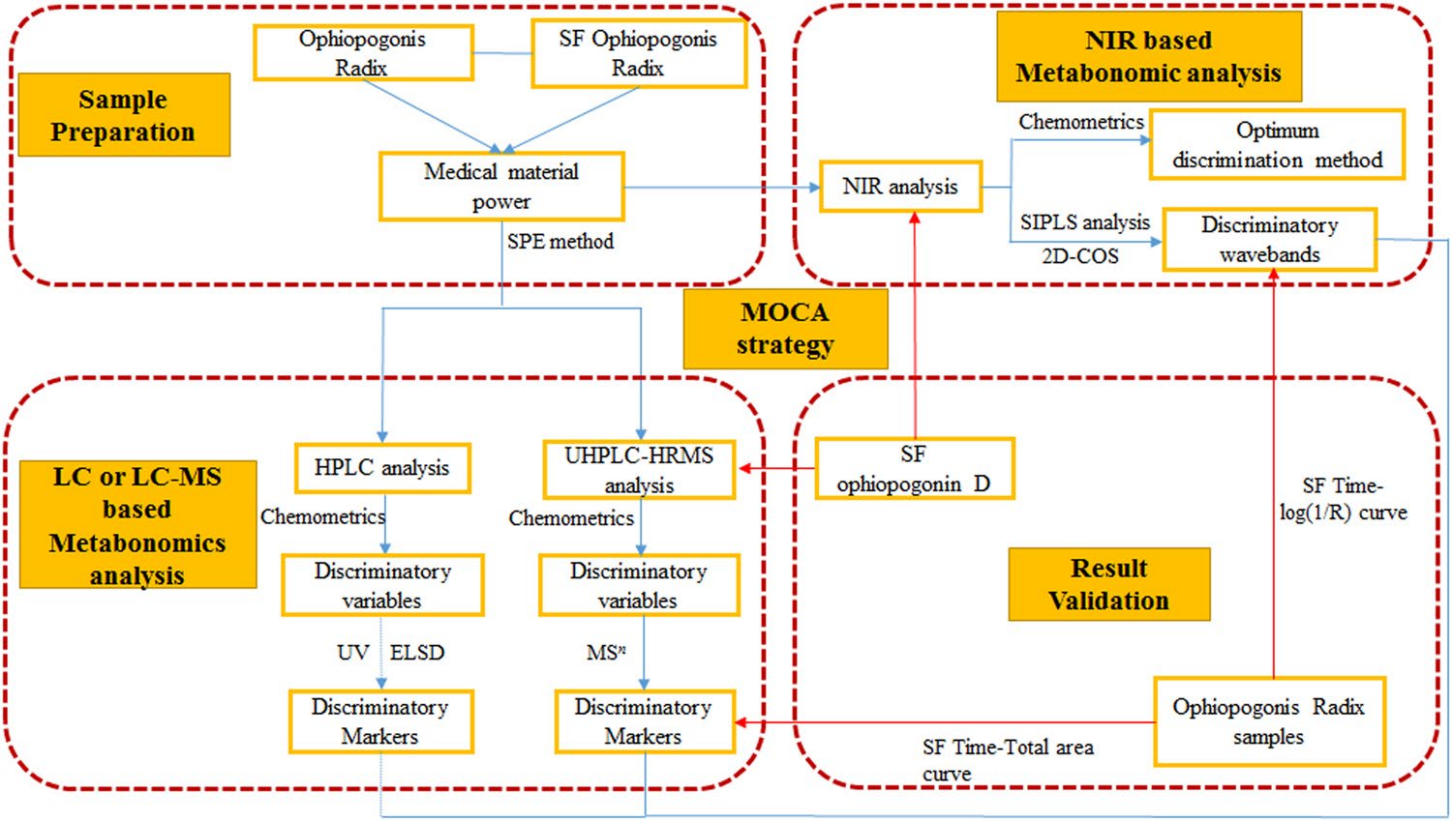
Summary diagram of the developed strategy and methodology.

Ophiopogonis Radix (Fig. 1), which belongs to Liliaceae family, is derived from the tuberous root of *Ophiopogon japonicus* (L. f.) Ker-Gawl. It is a common tonic product in China, Japan, and Korea^26^. Modern chemical, pharmacological, and therapeutic studies of Ophiopogonis Radix indicate that steroidal saponins and homoisoflavones are the major components which possess various bioactivities^27–30^. It should be noted that sulfur fumigation in the drying process of Ophiopogonis Radix is still frequently misused until now. Therefore, in present study, a novel and efficient strategy to rapidly discriminate sulfur-fumigated (SF) from none-sulfur-fumigated (NSF) Ophiopogonis Radix based on the established MOCA strategy was established. It was characterized by the multiple data-acquisition means including NIR, HPLC-DAD/ELSD, UHPLC-HRMS coupled with various multivariate statistical analysis methods, the summary diagram of presently developed analytical strategy and methodology was shown in Fig. 2.

## Results

### Discrimination of SF and NSF samples using NIR based metabonomics analysis

The NIR spectra (4000–10000 cm^−1^) for 26 batches of collected samples were illustrated in Fig. S1. Several weak absorption peaks were demonstrated in the second overtones region (SCOT, 10000–7100 cm^−1^) of the fundamental C-H stretching bands, while more fluctuations in the region of first combination-overtone (FCOT, 7100–4900 cm^−1^) and combination region (CR, 4900–4000 cm^−1^) were observed.

**Figure 3:**
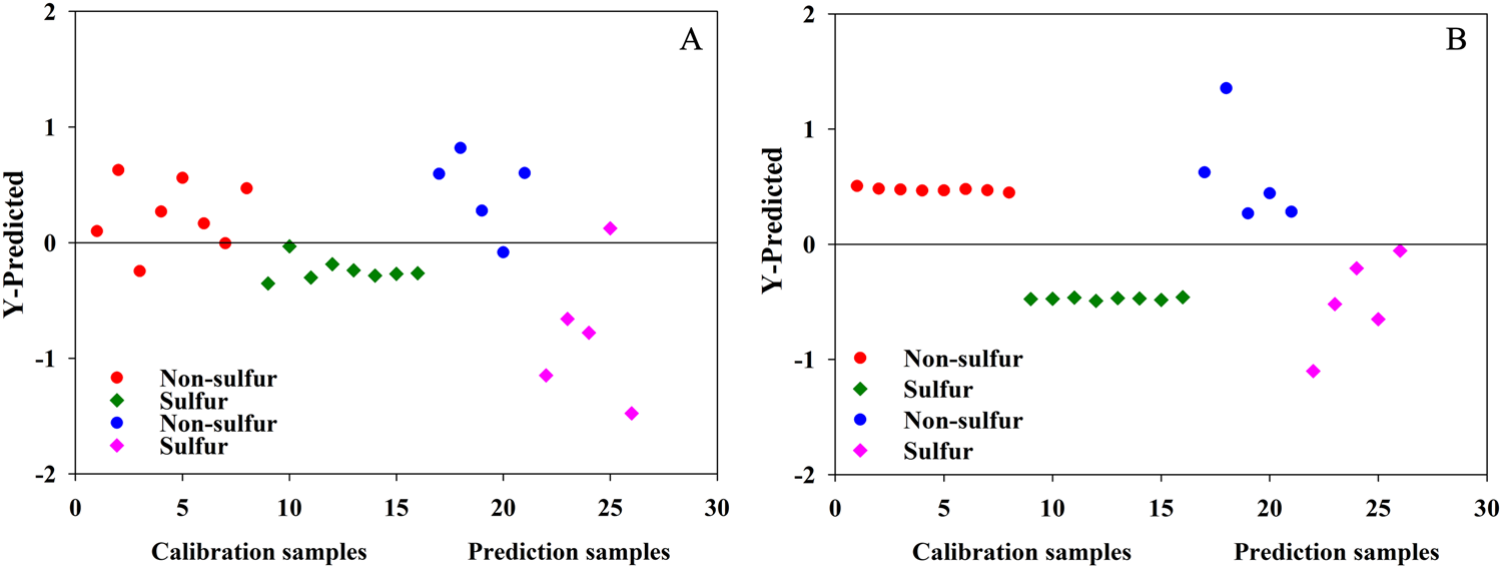
The discriminatory information in the raw spectra (Fig. 3A) and preprocess method of SG9+1st (Fig. 3B) for PLS-DA model.

It is well known that light scattering as well as the physical properties of samples, such as particle size, density, *etc*, could affect the raw spectra significantly. As a result, the phenomenon of spectral overlap and baseline drift was observed, resulting in no clear discriminatory information in the raw spectra from the PLS-DA analysis (Fig. 3A). Therefore, various data preprocess methods with different capability were utilized to reduce or even eliminate the influence of irrelevant information in the NIR spectra. Meanwhile, several parameters were computed to evaluate the established PLS-DA model based on preprocessed data, the root mean square error for cross-validation (RMSECV), the root mean square error for calibration (RMSEC), and the root mean square error for prediction (RMSEP), respectively. The smaller the value obtained, the better the model would be. Some additional parameters were also used to assess the accuracy of PLS-DA model, such as sensitivity (Se), specificity (Sp), and total accuracy^31, 32^, which were usually employed to measure the model ability in correctly classifying samples.

Our results demonstrated that the best data preprocessing method for discriminating SF from NSF Ophiopogonis Radix was Savitzky-Golay smoothing with 9 points plus first-order derivatives (SG9+1st), which could eliminate redundant information and manifest differences among samples (Table S1) to the highest degree. No clear separation between SF and NSF samples was obtained using the established PCA model based on the preprocess method of SG9 + 1st. However, the predicted Y values for the calibration and validation datasets were plotted to visualize the classification performance of PLS-DA model (Fig. 3B). As a result, most samples were successfully classified with the high percentages of Se, Sp, and total accuracy. (Figures for other preprocess methods were showed in the supplementary file, Fig. S2).

### Discrimination of SF and NSF samples using HPLC-DAD/ELSD based metabonomics analysis

Homoisoflavonoids in Ophiopogonis Radix possessed strong ultraviolet absorption, which could be predestined by HPLC-DAD at 296 nm. Meanwhile, ELSD was a more sensitive technique for the detection of steroidal saponins than DAD due to their weak ultraviolet absorptions. Accordingly, metabonomics analysis based on HPLC-DAD/ELSD was conducted after the optimization of chromatographic condition including mobile phase, flow rate, and formic acid addition, *etc*. Fig. S3 showed the representative chromatograms obtained from HPLC-DAD/ELSD analysis (Fig. S3A for ELSD, Fig. S3B for DAD at 296 nm). After peak alignment and removal of missing values, a total of 72 distinct features, which were selected based on the relative peak areas (≥1%), were obtained for subsequent data-processing.

At first, the principal factorial plane summarized 74.60% of the whole variability after the preprocessing method of mean standardization, but 26 batches of Ophiopogonis Radix could not be explicitly clustered into two groups. Therefore, supervised projections to latent structures PLS–DA model was then constructed and validated based on the training and test data sets to perform the discrimination. The PLS-DA model (Fig. S4A) resulted in a clear separation of SF and NSF samples with *R* ^2^(*Y*) of 81.9% and *Q* ^2^ of 61.1%, which demonstrated the model is statistically significant.

Furthermore, to guard against model over-fitting, permutation tests with 200 iterations (Fig. S4B) were performed in the PLS-DA model^33–35^. In general, statistical models are considered as statistical significant when the corresponding *Q* ^2^-intercept (−0.0701) value for permutation model is negative and the permuted *R* ^2^-value (0.4125) is lower than the original point of *R* ^2^-value (0.8188)^36^. Additionally, CV-ANOVA (analysis of variance testing of cross-validated predictive residuals) tests were used to confirm that two groups identified by PLS-DA are significantly different based on analytical data included in model^37, 38^. The common practice is to interpret a *p* value (0.024) lower than 0.05 as donating a significant model^39^.

In order to focus on the differential variables associated with samples separation, a set of components that made large contributions to the prediction of the sample were selected. First, significant original variables were extracted from the S-plot, which imaged both the covariance and correlation between the components and the modeled class, thus the risk of false positives in components selection was reduced. The markers fulfilled three different criteria: position in S plot (*p* > 0.05 and *p* (corr) > 0.3)^40–42^ (Fig. S4C), VIP value (>1.5) and t test (*p* < 0.05), and then 25 peaks were chosen as potential markers.

Nevertheless, most of the chromatographic peaks simultaneously meeting with the abovementioned three characteristics were too weak to obtain their real UV characteristic spectra. Moreover, the deficiency of corresponding reference standards and relevant database made it difficult to identify and confirm the structures of these discriminatory markers.

### Discrimination of SF and NSF samples using UHPLC-LTQ-Orbitrap HRMS based metabonomics analysis

**Figure 4:**
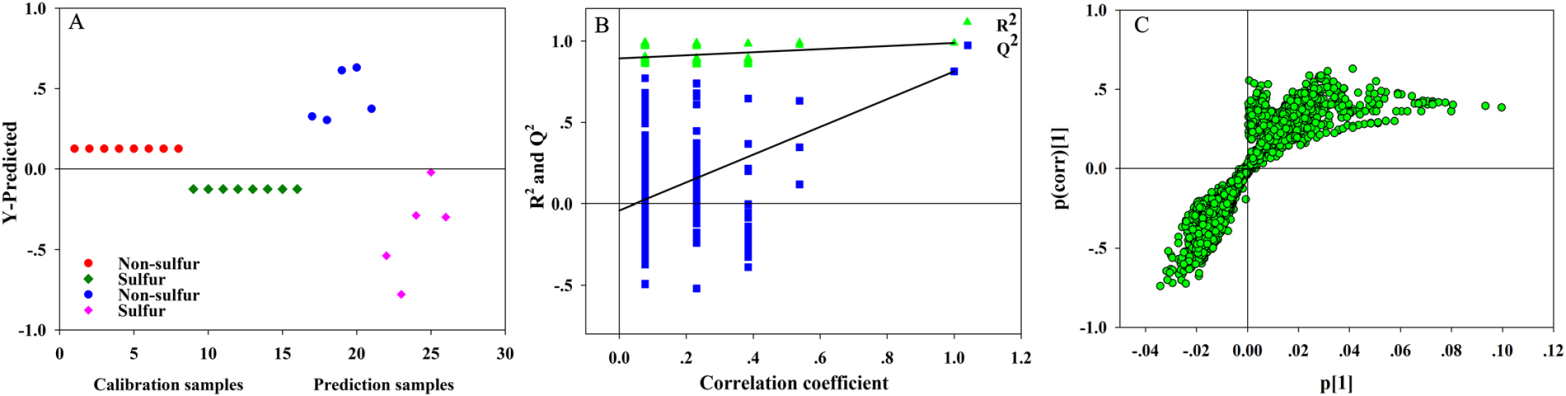
(**A**) The PLS-DA model of Ophiopogonis Radix using the UHPLC-HRMS data; (**B**) The PLS-DA model was considered as valid significantly since the corresponding Q2-intercept value is negative; (**C**) S-plot based on the UHPLC-HRMS data.

In order to make up to the drawback of HPLC-DAD/ELSD analysis, UHPLC-LTQ-Orbitrap HRMS instrument was employed to discriminate the SF and NSF samples. Fig. S5 showed the typical total ion current chromatograms (TIC) of one NSF and corresponding SF samples. As for the UHPLC-HRMS analysis, classification trends among the two types of Ophiopogonis Radix were observed using PCA although the trends were not exactly clear. Then, the PLS-DA model (Fig. 4A) constructed with *R* ^2^(*Y*) of 98.9% and *Q* ^2^ of 81.4% which described the calibration set well and also function as a predictive model. Results of the permutation tests with 200 iterations (Fig. 4B) showed *Q* ^2^-intercept value for permutation model was negative (−0.0301) and the permuted *R* ^2^-value (0.8896) was lower than the original point of *R* ^2^-value (0.9886). *p* value of the CV-ANOVA tests was 3.64 × 10^−4^, indicating the model was statistically significant and the results were reliable.

### Identification of differential chemical constituents

The constituents detected by UHPLC-LTQ-Orbitrap HRMS and fulfilled the three criteria that are S plot (*p* > 0.05 and *p* (corr) > 0.3), VIP value (>1.5) and t test (*p* < 0.05) were considered as the most relevant chemicals in discrimination of these two groups (Fig. 4C was the S-plot figure). As a result, a total of 78 chromatographic MS peaks were preliminarily screened. Because the peak areas of most screened discriminatory variables were too small to obtain their MS^*n*^ data, parent ion list-dynamic exclusion (PIL-DE) based data-acquisition method was utilized to accomplish the comprehensive acquisition of HRMS^1^ and MS^*n*^ data sets, which supplied great help to the following structural identification^21^.

**Figure 5:**
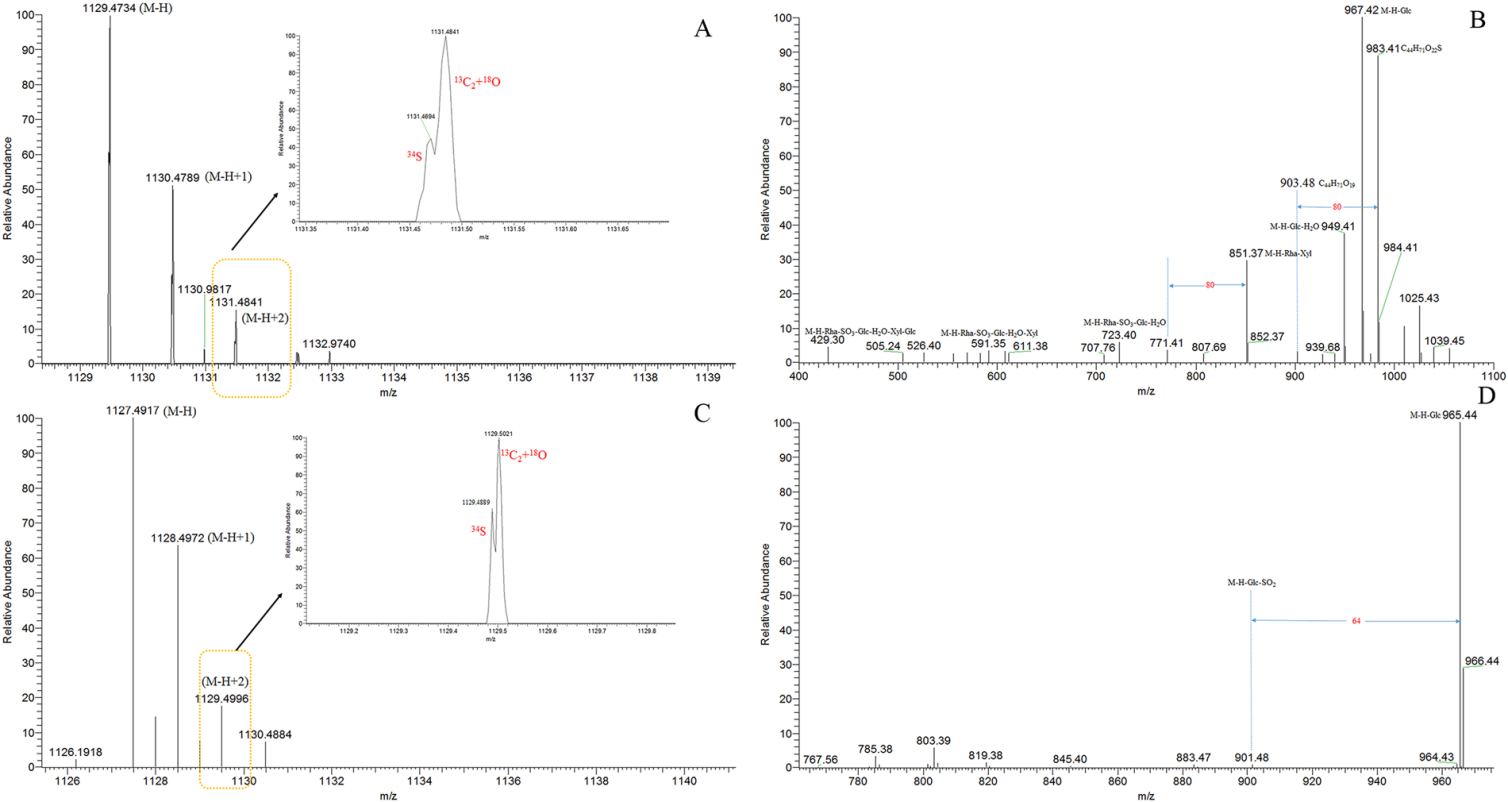
The mass fragmentation behaviors of identified markers (**A**) HRMS^1^ spectrum of No. 11; (**B**) ESI-MS^2^ spectrum of No. 11; (**C**) HRMS^1^ spectrum of No. 20; (**B**) ESI-MS^2^ spectrum of No. 20).

It was reported that those sulfur-containing saponins might be in the form of sulfate or sulfite^43^, which could be further confirmed by the isotopic pattern between ^34^S and ^13^C_2_+^18^O^44^. For example, marker **13** yielded its deprotonated molecule [M-H]^−^ ion at *m/z* 1129.4729 (C_50_H_81_O_26_S, error −0.20 ppm). In its ESI-MS^2^ spectrum, the diagnostic product ions at *m/z* 983, *m/z* 903, *m/z* 723, *m/z* 591, and *m/z* 429 were proposed to be generated through successive neutral losses of rhamnosyl moiety, sulfur moiety, glucosyl moiety, xylosyl moiety, and another glucosyl moiety in order. Among them, the product ions at *m/z* 983 and *m/z* 903 further indicated that the introduction of sulfate moiety to steroidal saponin molecule, which has not been ever reported before (Fig. 5B). Finally, marker **13** was tentatively identified as xylopyranose (1 → ?) ophiopojaponin B-sulfate or its isomers. Marker **9** generated its [M-H]^−^ ion at *m/z* 1127.4939 (C_51_H_83_O_25_S, error 0.03 ppm). The product ions at *m/z* 965, *m/z* 901, *m/z* 755, *m/z* 593, *m/z* 575, and *m/z* 413 were yielded through successive neutral losses of glucosyl moiety, sulfite moiety, rhamnosyl moiety, glucosyl moiety, H_2_O, and glucosyl moiety, orderly (Fig. 5D). The characteristic product ions at *m/z* 965 and *m/z* 901 probably validated the occurrence of sulfite moiety in a certain steroidal saponin molecule. Therefore, marker **9** was tentatively characterized as trigoneoside Iva-sulfite or its isomers. Their isotopic patterns of ^34^S and ^13^C_2_+^18^O were showed in Fig. 5A and C.

Likewise, according to the fragmentation behaviors, isotopic patterns, diagnostic product ions^45^, a total of 45 discriminatory markers were identified tentatively (shown in Table S2). Among them, 38 were assigned to be sulfate/sulfite derivatives of the reported steroidal saponins, and 9 were steroidal saponins. Among the other 31 screened differential peaks, the contents of 10 peaks were too low to obtain their MS^*n*^ stage data, 12 peaks could not be attributed to steroidal saponins based on their HRMS^1^ data, and the other 9 peaks cannot be elucidated due to the lack of relevant database.

### MOCA analysis based on NIR, HPLC, and LC-MS metabonomics analysis

**Figure 6:**
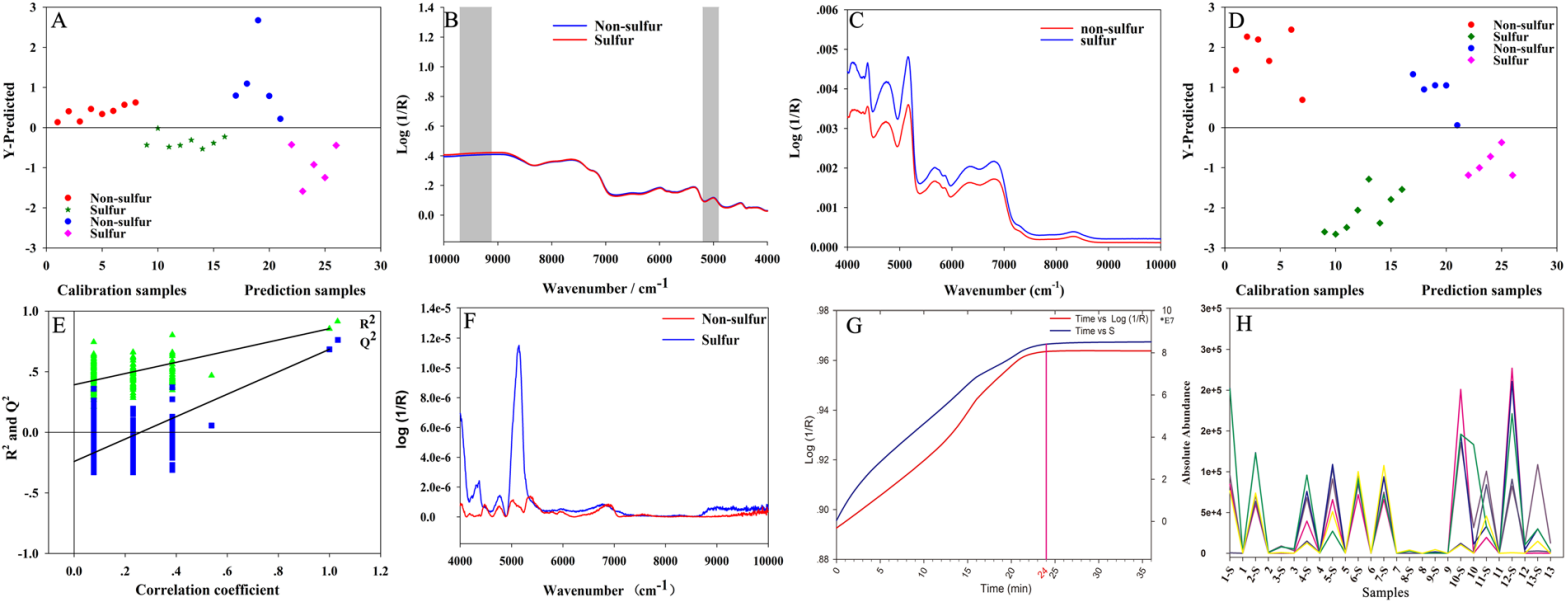
(**A**) The discrimination information of PLS-DA model combined by the preprocessing method of wavelet denosing of spectra; (**B**) The specific wavebands screened by the SiPLS; (**C**) The synchronous spectra of NSF and SF Ophiopogonis Radix. (**D**) PLS-DA model of Ophiopogonis Radix using the identified biomarkers data; (**E**) The PLS-DA model was considered as valid significantly since the corresponding Q2-intercept value is negative; (**F**) The synchronous spectra of NSF and SF of Ophiopogonin D; (**G**) Log (1/R)-Time curve of NIR analysis; (**H**) Total peak areas of these elucidated 38 compounds - Time curve.

*Screening of NIR specific wavebands*. NIR spectrum is constructed by different wavebands, but not all of them possess the special discrimination ability. Therefore, the synergy interval partial least squares (SiPLS) with 3 intervals were employed to screen the potential wavebands which indicated the significant differences between SF and NSF samples. In general, the latent factor could be set from 1 to 10 to avoid over-fitting, and the SiPLS with spectroscopic transformation would be the best discriminant model (Table 1). As shown in Fig. 6A, samples were properly classified *via* the established model and all the Se, Sp, and accuracy parameters reached 1.0, which indicated good model performance. Moreover, the special wavebands including 4902–5200 and 9114–9704 cm^−1^ (Fig. 6B) was screened, which might represent the special structural characteristic of the discriminatory markers.

**Table 1:** The PLS-DA model of different preprocessing methods for screening the specific wavebands by SiPLS.

	LVs	Model evaluation			Discriminant results		
		RMSECV	RMSEC	RMSEP	Se	Sp	Accuracy
Raw*	5	0.6459	0.4452	1.1014	0.8	1	0.9
MSC	15	0.527	4.02E-13	1.5248	0.6	0.4	0.5
SNV	14	0.5487	8.06E-06	1.5012	1	0.6	0.8
Baseline	1	1.0358	0.9997	0.9948	0.4	0.6	0.5
Normalization	4	0.6899	0.5948	0.6705	0.6	1	0.8
S-T	4	0.6365	0.5019	0.6748	1	1	1
WDS	11	0.5436	0.0254	0.4097	1	1	1
S-G(9) + 1^st^	4	0.696	0.5597	0.8499	1	0.8	0.9
S-G(11) + 1^st^	11	0.6004	0.003	0.7203	0.6	1	0.8
S-G(9) + 2^nd^	1	1.0057	0.6242	0.8726	0.8	0.6	0.7
S-G(11) + 2^nd^	1	1.0218	0.7848	0.8603	1	0.8	0.9

^*^A variety of preprocessing methods to extract the useful information from noise for the spectroscopic data were compared, such as multiplicative scatter correction (MSC), standard normal variate transformation (SNV), baseline, normalization, spectroscopic transformation (ST), wavelet denosing of spectra (WDS), Savitzky-Golay smoothing with 9 points (SG(9)) plus first-order derivatives, SG(9) plus second-order derivatives, SG(11) plus first-order derivatives, and SG(11) plus second-order derivatives.

Subsequently, a two-dimensional correlation spectrum (2D-COS) method was further employed to explain the screened wavebands. Figure S6 is the 2D-COS spectra of the NSF and SF sample, and a special waveband (Fig. 6C) of SF samples emerged in the zone where the strongest autocorrelation peak appeared when compared with NSF samples. The screened wavenumber was around 5000 cm^−1^, which was in accordance with the screened wavebands by SiPLS method. According to previous report^46^, S-H and S-OH perssad represented a strong absorption in the wavenumber of 5000–5200 cm^−1^. Therefore, a positive confirmation can be made that there was a close relationship between the screened wavebands and the sulfur-containing markers in the SF samples.

#### *Explanation of the special wavebands from UHPLC-LTQ-Orbitrap HRMS perspective*

In the UHPLC-HRMS based metabonomics experiment, 38 sulfur-containing markers were finally screened and identified according to the S-plots of PLS-DA model based on the UHPLC-LTQ-Orbitrap HRMS data (Table S2). Although PCA analysis failed to identify groups, a degree of clustering was observed, which showed better performance than the original PCA model. Meanwhile, PLS-DA generated a scores plot that showed a clear distinction between two groups (Fig. 6D) with statistically significant model (*R* ^2^(Y) = 0.856, *Q* ^2^ = 0.714). Besides, the statistical validities of the PLS-DA model were also assessed by performing rigorous permutation tests with 200 permutations (Fig. 6E). The results showed *Q* ^2^-intercept of the permutation model was negative (−0.2410) and the permuted *R* ^2^-value (0.3928) was lower than the original point of *R* ^2^-value (0.8560), which indicated the model was reliable. Moreover, *p* value of the CV-ANOVA tests was 2.27 × 10^−4^, which further confirmed the model was eligible. The well-fitted PLS-DA model constructed by the 38 identified markers was the contributing factor to the changes in the NIR spectrum.

#### *Validation using ophiopogonin D and the established MOCA strategy*

Many other influences would cause some negative results during the SF process due to the complicated constituents property of Ophiogonis Radix. Therefore, to validate the above results, a reference standard of the main representative steroidal saponins chosen from the 38 identified markers, namely ophiopogonin D, was sulfur-fumigated and analyzed using the same methods. The autocorrelation curve (Fig. 6F) of the SF and NSF of the ophiopogonin D was intercepted from the 2D-COS spectra which were presented in Figs S7A and S7B. Obvious difference between SF and NSF ophiopogonin D in the wavebands of 5000 cm^−1^ was observed, which was also in accordance with the screened wavebands by SiPLS model. The subsequent UHPLC-HRMS analysis of SF ophiopogonin D mixture also indicated the newly generated constituents (Fig. S8B) besides ophiopogonin D (Fig. S8A) during the process of sulfur-fumigated. The structure of these new emerged peaks was identified rudimentary as sulfate-derivatives of ophiopogonin D based on the HRMS data, which were 79.95 (SO_3_) Da more than that of standard reference.

#### *Validation based on the accumulation of sulfur-containing components by the established MACO analysis*

An accumulative effect of the identified representative sulfur-containing compounds was observed with prolonged SF time. From the Log (1/R)-Time curve (Fig. 6G) of NIR analysis, the total absorption value of those sulfur-containing compounds in 5000 cm^−1^ also showed an increasing trend along with prolonged SF time. After 24 h, the absorption value reached a plateau. Meanwhile, a similar trend was also observed for the total peak areas of these elucidated 38 markers versus SF time curve (Fig. 6H). It also indicated that the results of MOCA were reliable and credible in the discrimination of SF Ophiopogonis Radix.

## Discussions

Sulfur fumigation is becoming a controversial topic recently due to its potential damages to CHM, among which, chemical constituent transformation is the dominant one. NIR, HPLC, and LC-MS based research on SF of CHM have been developed and applied for quality control for decades. However, the data obtained by single detection method is not adequate to provide an in-depth explanation of the mechanism of sulfur fumigation. In this study, a widely used Chinese medicinal herb Ophiopogonis Radix was adopted as an example. Multi-omics and multi-variate analysis based MOCA was viewed as strategy to make a correlation analysis of obtained NIR spectra, HPLC chromatograms, and HRMS data. MOCA not only could be adopted to unambiguously discriminate SF Ophiopogonis Radix from NSF ones but also could clarify the inherent mechanism of NIR judgment method when it was purposefully linked to HPLC and UHPLC-MS analysis. This study established a rapid and sensitive method for the discrimination of SF Ophiopogonis Radix, which is definitely beneficial to the quality control of CHM.

Among the various detection technologies applied in the established MOCA strategy in this study, NIR based metabonomics coupled with data-preprocessing method (SG9 + 1st) and chemometrics (PCA and PLS-DA) provided an effective technical assistance to discriminate SF Ophiopogonis Radix from NSF ones. In order to reveal the chemical transformation information, UHPLC-LTQ-Orbitrap MS was also utilized. However, the new generated sulfur-containing derivatives during sulfur fumigation process were usually at low concentration, hence their MS signals often being masked by the inherent constituents showed relatively high concentrations. To solve this problem, UHPLC chromatographic column that possessed smaller particle size (1.7 μm), narrower peak shape, higher resolution, *etc*, coupled with a relative long separation time (up to 100 min) was developed to achieve better separation of the sulfur-containing derivatives. In addition, the special scan approach and data-dependent acquisition method built in LTQ-Orbitrap MS also provided MS^1^ (<5 ppm) with high resolution, scan efficiency (a full-scan mass spectrum acquired with a mass resolution of 30,000 for Orbitrap only needs 0.4 s, and provides 25 data points across a peak of width at baseline of 10 s), and abundant MS^*n*^ data sets, which ascertained a rapid and accurate identification of discriminatory markers of LC-MS based metabonomics.

Meanwhile, various pattern recognition methods were investigated for the rapid discrimination. Usually, PCA is well established exploration tool for preliminary study of trends, groupings and outliers in high dimensional data, especially when the number of variables exceeds the sample size considerably. However, the concentration of new generated constituents was too low to be detected unless by UHPLC-MS technology which means that their relatively small peak areas may cause the failure PCA classification. Whereas if PLS-DA was applied additionally, its X matrix contained the NIR, HPLC-DAD/ELSD, and UHPLC-MS variables while Y matrix contained the class information for each sample. Therefore, this kind of supervised pattern recognition method could greatly improve the classification precision and accuracy in the present experiment.

What’s more, it was noticeable that only identified steroidal saponin sulfate/sulfite derivatives played vital roles in the discrimination of SF Ophiopogonis Radix. Since homoisoflavones are another important category of constituents, artificially designed homoisoflavone sulfate/sulfite derivatives (HSDs) were created for the first time (Y = X + SO_3_ or SO_2_, X: the accurate molecular weights of known homoisoflavons, Y: the accurate molecular weights of HSDs). Then, their existence was ascertained using high-resolution extracted ion chromatograms (HREICs). Results showed that these potential HSDs did exist in both SF and NSF samples, and their concentrations did not change evidently during sulfur fumigation process. As a matter of fact, as illustrated in Fig. 6H, the concentration of these discriminatory steroidal saponins markers was not zero in NSF samples. This phenomenon might be attributed to the application of sulfur-containing fertilizers, pesticides, and the sulfide exited in polluted air, soil, and water. However, during the sulfur fumigation process, steroidal saponins were converted to sulfate/sulfite derivatives much readily than homoisoflavones, the mechanism of which needs further study.

In conclusion, our result revealed the intrinsic mechanism of discriminatory NIR waveband by means of UHPLC-HRMS analysis for the first time. The established MOCA strategy also provided a promising NIR based differential method for SF Ophiopogonis Radix, which could be exemplary for future researches on the rapid discrimination of other SF CHM.

## Methods

### Sample collection and preparation

13 batches (Table S3) of NSF Ophiopogonis Radix were purchased from co-operated farmers from several different authenticated regions in China. The quality of obtained samples was checked using acid distillation-iodimetric titration method proposed by Chinese pharmacopoeia (version 2015). The SF Ophiopogonis Radix samples were prepared following the modified procedures similar to that performed by illicit wholesalers^43, 47^. The voucher specimens were deposited in Beijing Research Institute of Chinese Medicine, Beijing University of Chinese Medicine, China. Meanwhile, the SF ophiopogonin D utilized for validation was also prepared with same methods.

SF Ophiopogonis Radix sample (3.0 g) and NSF sample (3.0 g) were accurately weighted and then ultrasonic-extracted with 70% methanol (25 mL) for 30 min. The supernatants were then filtered and steamed (50 °C) nearly dry. The residue was redissolved in water (2 mL) and loaded to preprocessed SPE columns, which were sequentially washed with water (3 mL) and methanol (3 mL). The methanol eluates were collected and filtered by a 0.22 μm PTFE syringe filter. To ensure the HPLC and LC-MS based metabonomics data quality, pooled quality control (QC) samples were prepared by mixing equal amounts of 26 sample solutions. In addition, Ophiopogonin D and SF Ophiopogonin D were weighed then dissolved in methanol to produce reference solutions (0.1 mg/mL), the reference solutions were then stored under 4 °C prior to analysis.

### Data-acquisition

#### NIR based analysis

The NIR spectra were collected in the integrating sphere diffuse reflectance mode with Antaris Nicolet FT-NIR system (Thermo Fisher Scientific Inc., USA). Each spectrum was an average of 32 scans with the resolution of 8 cm^−1^ between 10,000 and 4,000 cm^−1^ at ambient temperature. The spectra were recorded as reference absorbance values in air. Each sample was scanned three times, and the final spectrum used for model building was the average of three measurements.

#### HPLC-DAD/ELSD based analysis

The chromatographic separation was achieved on a DIONEX Ultimate 3000 UHPLC system (Thermo Fisher Scientific, MA, USA) using Zorbax SB-Aq C18 column (4.6 × 150 mm, 3.5 μm; Agilent, USA) at room temperature. A gradient elusion program was conducted for chromatographic separation at 1.0 mL/min with solvent A (0.1% formic acid) and solvent B (acetonitrile/methanol = 3:1, *v/v*) as follows: 0–3 min, 5–10% B; 3–6 min, 10–21% B; 6–18 min, 21–26% B; 18–40 min, 26–51% B; 40–58 min, 51–55% B; 58–60 min, 55–72% B. The UV detection wavelength was set at 296 nm, while ELSD detection parameters were set as follows: drift tube temperature of 100 °C and gas flow rate of 2.7 L/min. All samples were randomly coded and subjected into the HPLC system, whose stability was validated by running the QC sample every 10 samples during the data-acquisition process.

#### UHPLC-LTQ-Orbitrap MS based analysis

The chromatographic separation was carried out on a DIONEX Ultimate 3000 UHPLC system using an ACQUITY UHPLC HSS T3 column (100 mm × 2.1 mm, 1.8 μm; Waters Corp., Milford, MA, USA) at 25 °C. The mobile phases were same as HPLC analysis with a gradient program set as follows: 0–2 min, 10% B; 2–10 min, 10–20% B; 10–28 min, 20–26% B; 28–31 min, 26% B; 31–45 min, 26–32% B; 55–90 min, 46–67% B; 90–93 min, 67–80% B; 93–95 min, 80% B. The flow rate was 0.30 mL/min and the injection volume was 2 μL. The routine MS detector was were provided in the Online Appendix. Especially, the screened potential marker ions were added into PIL (dynamic excluded other irrelevant ions) to obtain specific MS^*n*^ datasets. In addition, an ultra-high-resolution of mass spectrometry (100,000 FWHM @ 400 *m/z*) was employed to validate the identified sulfur derivatives in full scan mode. The QC sample was run every 10 samples to study the LC-MS system stability.

### Validation

Five concentration levels of ophiopogonin D were used to perform SF process as samples and four non processing concentration levels as references. SF ophiopogonin D samples (0.5, 1.0, 2.0, 2.5, and 5.0 mg) were weighed, and each sample was then thoroughly mixed with 5.0 mg dextrin. Meanwhile, ophiopogonin D samples (0.5, 1.0, 1.5, and 2.0 mg) were prepared in the same way for economic consideration without influencing reliability of the results. Those nine samples were analyzed using the same NIR analysis, whereas the gradient elusion program in the LC-MS analysis was set as follows: 0–22 min, 80–20% B; 22–23 min, 20–80% B while the other parameters maintained.

At the same time, one batch of NSF sample (JSPACM-05–1) was randomly chosen for observation of the accumulative effects of those discriminatory sulfur-containing markers during the sulfur-fumigated process. Different SF samples at 0, 4, 12, 16, 20, 24, and 36 h from the beginning of SF process were obtained and analyzed using the same NIR and LC-MS method mentioned above. Finally, the correlation between the total area of 38 discriminatory markers and the NIR absorption at 5000 cm^−1^ was determined.

### Software

Thermo Xcalibur 2.1 workstation (Thermo Scientific, Germany) was adopted to acquire and process the ESI-MS/MS data. Their normalization was accomplished using Sieve 2.1 (Thermo Scientific, USA) software, which referred to background subtraction, component detection, and peak alignment. SIMCA-P + (version 11.5, Umetrics, Sweden) and Unscrambler 7.0 (CAMO, Norway) software was utilized to carry out the spectral pretreatments. Prior to model construction, the original data set was divided into a calibration set and a validation set using the Kennard-Stone algorithm; 16 of the 26 samples were included in the calibration set and the other 10 samples were included in the validation set. Matlab 2009a (The MathWorks, Inc., USA) software and the PLS toolbox were used for the PLS-DA. Other data analysis was performed using in-house routines programmed in Matlab software. The iToolbox used to run SiPLS algorithms was downloaded from http://www.models.kvl.dk/ for the wavelength selection.

## Electronic supplementary material

Supplementary  information
