# Supplementary material for: Novelty application of multi-omics correlation in the discrimination of sulfur-fumigation and non-sulfur-fumigation Ophiopogonis Radix
Source: Sci Rep. 2017 Aug 30;7:9971. doi: 10.1038/s41598-017-10313-1 (PMC5577285; doi:10.1038/s41598-017-10313-1)

Supporting Information

Novelty application of multi-omics correlation in the discrimination of sulfur-fumigation and non-sulfur-fumigation Ophiopogonis Radix

Shengyun Dai[1,+], Zhanpeng Shang[1,+], Fei Wang[1], Yanfeng Cao[1], Xinyuan Shi[1], Zhaozhou Lin[2], Zhibin Wang[3], Ning Li[4], Jianqiu Lu[1,*], Yanjiang Qiao[1,*] & Jiayu Zhang[3,*]

[1] *School of Chinese Pharmacy, Beijing University of Chinese Medicine, Beijing 100102, China*

[2] *Beijing Hospital of Traditional Chinese Medicine, Capital Medical University, Beijing 100010, China*

[3] *Beijing Research Institute of Chinese Medicine, Beijing University of Chinese Medicine, Beijing 100029, China*

[4] *Shenzhen Research Institute, The Hong Kong University of Science and Technology, Shenzhen 518057, China*

[+]: These authors contributed equally to this work.
[*]: Correspondence and requests for materials should be addressed to J.L. (email: Lujq@vip.sina.com) or Y.Q. (email: yjqiao@263.net) or J.Z. (email: zhangjiayu0615@163.com)

Chemicals and materials. Ophiopogonin D (purity ≥ 98%) was purchased from Chengdu Biopurify Phytochemical Ltd (Chengdu, China). The voucher specimens of Ophiopogonis Radix were deposited in Beijing Research Institute of Chinese Medicine, Beijing University of Chinese Medicine, China. Chromatographic solvents of LC-MS grade including acetonitrile, methanol and formic acid were purchased from Fisher Scientific (Fisher, NJ, USA). All the other chemicals of analytical grade were purchased from Beijing Chemical Works (Beijing, China). Grace Pure™ SPE C18-Low solid-phase extraction cartridges (200 mg/3 mL, 59 μm, 70 Å) were purchased from Grace Davison Discovery Science™ (Deerfield, IL, USA). Ultra-pure Milli-Q water (Milliope, Billerica, MA, USA) was used for the preparation of all solutions.

LTQ-Orbitrap mass spectrometer parameters. The LTQ-Orbitrap mass spectrometer was connected to the UHPLC system *via* an electrospray ionization (ESI) interface in negative mode. The mass range was set at *m/z* 100-1800 Da at a resolving power of 30,000. The optimized operating parameters were set as follows: capillary voltage of 35 V, electrospray voltage of 3.0 kV, capillary temperature of 350℃, sheath gas flow rate of 40 (arbitrary units), auxiliary rate of 20 (arbitrary units), and tube lens of 110 V. Data-dependent ESI-MS^2 analyses were triggered by the three most-abundant ions from the precursor ions. Collsion-induced dissociation (CID) was performed with an isolation width of 2.0 Da. The collision energy was set to 35%.

Table S1. The PLS-DA model of different preprocessing methods.

	LVs	Model evaluation			Discriminant results		
		RMSECV	RMSEC	RMSEP	Se	Sp	Accuracy
Raw	5	0.7055	0.6522	0.6018	0.8	0.8	0.8
MSC	6	0.6921	0.2867	0.9433	0.4	0.8	0.6
SNV	6	0.6924	0.4921	0.8952	0.4	0.8	0.6
Baseline	5	0.6928	0.6403	0.6550	0.6	1	0.8
Normalization	6	0.7092	0.4493	0.7441	0.6	0.8	0.7
S-T	4	0.7114	0.5929	0.6350	1	0.8	0.9
WDS	5	0.6983	0.3911	1.3553	0.4	0.6	0.5
S-G(9)+1st	8	0.5896	0.0269	0.5924	1	1	1
S-G(11)+1st	11	0.6257	0.0533	0.6524	1	0.8	0.9
S-G(9)+2nd	2	0.8659	0.4939	0.7705	0.8	0.8	0.8
S-G(11)+2nd	6	0.7963	0.0135	0.6988	0.8	0.8	0.8

Table S2. The predicted elemental compositions, experimental mass, and characteristic fragment ions of identified markers.

No	VIP values	Formula	t_R	Relative intensity	Experimental mass *m/z*	MS/MS fragment ions	Identification
1	7.28	$C_{45}H_{73}O_{24}S$	25.09	1.07×10^{7}	997.4282	MS^2[997]: 979[M-H-H$_2$O]$^-$, 851[M-H-Rha]$^-$, 835[M-H-Glc]$^-$, 671[M-H-Rha-Glc-H$_2$O]$^-$	ophiopojaponin B/isomer-sulfate or ?-hydroxy-protobioside/isomer-sulfate
2	6.06	$C_{45}H_{71}O_{18}$	76.30	9.96×10^{6}	899.4640	MS^2[899]: 853[M-H-HCOOH]$^-$, 721[M-H-HCOOH-Xyl]$^-$, 707[M-H-HCOOH-Rha]$^-$	Ophiopogonin D + HCOOH/isomer
3	5.37	$C_{45}H_{73}O_{24}S$	25.42	8.00×10^{6}	997.4279	MS^2[997]: 979[M-H-H$_2$O]$^-$, 851[M-H-Rha]$^-$, 835[M-H-Glc]$^-$, 817[M-H-Glc-H$_2$O]$^-$, 771[M-H-Rha-80]$^-$, 691[M-H-Rha-Glc-H$_2$O]$^-$	ophiopojaponin B/isomer-sulfate or ?-hydroxy-protobioside/isomer-sulfate
4	5.33	$C_{50}H_{79}O_{25}$	32.05	1.07×10^{6}	1079.5247	MS^2[1079]: 1061[M-H-H$_2$O]$^-$, 947[M-H-Xyl]$^-$, 933[M-H-Rha]$^-$, 917[M-H-Glc]$^-$	Ophiofurospiside I/isomer
5	5.19	$C_{45}H_{71}O_{20}$	58.31	3.38×10^{6}	931.4545	MS^2[931]: 785[M-H-Rha]$^-$, 623[M-H-Rha-Glc]$^-$, 461[M-H-Rha-Glc-Glc]$^-$	?-hydroxy-(25R)-14α,17α-hydroxyspirost-5-en-3β-yl 3-*O*-α-L-rhamnpyranosyl-(1→2)-β-D-glucopyranosyl-(1→3)-β-D-glucopyranoside/isomer
6	4.64	$C_{45}H_{73}O_{21}S$	27.26	4.74×10^{6}	981.4338	MS^2[981]: 835[M-H-Rha]$^-$, 819[M-H-Glc]$^-$, 801[M-H-Glc-H$_2$O]$^-$, 673[M-H-Rha-Glc]$^-$, 655[M-H-Rha-Glc-H$_2$O]$^-$	protobioside/isomer-sulfate or ophiopojaponin B/isomer-sulfite
7	3.76	$C_{47}H_{75}O_{23}S$	36.38	1.01×10^{6}	1039.4390	MS^2[1039]: 1021[M-H-H$_2$O]$^-$, 979[M-H-H$_2$O-42]$^-$, 877[M-H-Glc]$^-$, 859[M-H-Glc-H$_2$O]$^-$	Ac (1→?) ophiopojaponin B /isomer-sulfate
8	3.67	$C_{47}H_{75}O_{19}$	80.35	4.56×10^{5}	943.4745	MS^2[943]: 901[M-H-42]$^-$, 883[M-H-42-H$_2$O]$^-$, 755[M-H-42-Rha]$^-$	Ac-protobioside/isomer
9	3.60	$C_{51}H_{83}O_{25}S$	26.64	1.63×10^{6}	1127.4911	MS^2[1127]: 981[M-H-Rha]$^-$, 965[M-H-Glc]$^-$, 901[M-H-Glc-64]$^-$, 803[M-H-2Glc]$^-$, 755[M-H-Glc-64-Rha]$^-$, 593[M-H-2Glc-64-Rha]$^-$, 413[M-H-3Glc-64-Rha-H$_2$O]$^-$	trigoneoside Iva/isomer-sulfite or Xyl (1→?) ophiopojaponin B/isomer-sulfite

10	3.39	$C_{51}H_{83}O_{26}S$	22.09	9.07×10^{5}	1143.4861	MS^2[1143]: 981[M-H-Glc]$^-$, 917[M-H-Glc-64]$^-$, 889[M-H-Glc-64-H_2O]$^-$, 819[M-H-2Glc]$^-$, 801[M-H-2Glc-H_2O]$^-$, 771[M-H-Glc-64-Rha]$^-$, 591[M-H-2Glc-64-Rha-H_2O]$^-$	Glc-(1→?) ophiopojaponin B/isomer-sulfite
11	3.36	$C_{47}H_{75}O_{22}S$	32.98	1.05×10^{6}	1023.4428	MS^2[1023]: 1005[M-H-H_2O]$^-$, 963[M-H-H_2O-42]$^-$, 861[M-H-Glc]$^-$, 843[M-H-Glc-H_2O]$^-$, 801[M-H-Glc-H_2O-42]$^-$, 655[M-H-Glc-H_2O-42-Rha]$^-$	Ac (1→?) Xyl (1→?) 12-hydroxy ophiogenin 3-*O*-*α*-L-Rha-(1→2)-*β*-*D*-Glc/isomer-sulfate
12	3.13	$C_{51}H_{83}O_{24}$	49.15	5.95×10^{5}	1079.5269	MS^2[1079]: 1061[M-H-H_2O]$^-$, 947[M-H-Xyl]$^-$, 933[M-H-Rha]$^-$, 917[M-H-Glc]$^-$	Ophiofurospiside I/isomer
13	2.97	$C_{50}H_{81}O_{26}S$	25.31	8.70×10^{5}	1129.4701	MS^2[1129]: 1111[M-H-H_2O]$^-$, 983[M-H-Rha]$^-$, 967[M-H-Glc]$^-$, 949[M-H-Glc-H_2O]$^-$, 903[M-H-Rha-80]$^-$, 851[M-H-Rha-Xyl]$^-$, 771[M-H-Rha-Xyl-80]$^-$	Xyl (1→?) ophiopojaponin B/isomer sulfate
14	2.96	$C_{44}H_{69}O_{20}S$	59.58	4.76×10^{6}	949.4124	MS^2[949]: 803[M-H-Rha]$^-$, 787[M-H-Rha-H_2O]$^-$, 671[M-H-Rha-Xyl]$^-$	14-hydroxy-sprengerinin C/isomer-sulfate
15	2.92	$C_{51}H_{83}O_{27}S$	20.52	7.00×10^{6}	1159.4762	MS^2[1159]: 1013[M-H-Rha]$^-$, 997[M-H-Glc]$^-$, 915[M-H-Glc-64-H_2O]$^-$, 851[M-H-Glc-Rha]$^-$, 833[M-H-Glc-Rha-H_2O]$^-$, 817[M-H-Glc-Rha-2H_2O]$^-$, 751[M-H-Glc-64-H_2O-Rha]$^-$, 589[M-H-2Glc-64-H_2O-Rha]$^-$, 409[M-H-3Glc-64-2H_2O-Rha]$^-$	?-hydroxy Glc (1→?)-Glc (1→?) ophiogenin 3-*O*-*α*-L-Rha-(1→2)-*β*-*D*-Glc/isomer-sulfite
16	2.84	$C_{47}H_{75}O_{23}S$	30.00	9.12×10^{5}	1039.4373	MS^2[1039]: 1021[M-H-H_2O]$^-$, 979[M-H-H_2O-42]$^-$, 893[M-H-Rha]$^-$, 877[M-H-Glc]$^-$	Ac (1→?) ophiopojaponin B /isomer-sulfate
17	2.58	$C_{47}H_{75}O_{22}S$	39.11	9.11×10^{5}	1023.4421	MS^2[1023]: 1005[M-H-H_2O]$^-$, 963[M-H-H_2O-42]$^-$, 861[M-H-Glc]$^-$, 843[M-H-Glc-H_2O]$^-$, 801[M-H-Glc-H_2O-42]$^-$, 655[M-H-Glc-H_2O-42-Rha]$^-$	Ac (1→?) Xyl (1→?) 12-hydroxy ophiogenin 3-*O*-*α*-L-Rha-(1→2)-*β*-*D*-Glc/isomer-sulfate
18	2.57	$C_{50}H_{81}O_{26}S$	21.89	5.17×10^{5}	1129.4739	MS^2[1129]: 1111[M-H-H_2O]$^-$, 983[M-H-Rha]$^-$, 967[M-H-Rha-H_2O]$^-$, 949[M-H-Rha-2H_2O]$^-$, 903[M-H-Rha-80]$^-$, 851[M-H-Rha-Xyl]$^-$,	Xyl-(1→?) ophiopojaponin B/isomer sulfate

						771[M-H-Rha-Xyl-80]$^-$, 723[M-H-Rha-80-Glc-H_2O]$^-$, 591[M-H-Rha-Xyl-80-Glc-H_2O]$^-$, 429[M-H-Rha-Xyl-80-2Glc-H_2O]$^-$	
19	2.47	$C_{51}H_{83}O_{23}$	46.62	6.53×10^{5}	1063.5306	MS^2[1063]: 1045[M-H-H_2O]$^-$, 931[M-H-Xyl]$^-$, 917[M-H-Rha]$^-$, 901[M-H-Glc]$^-$	Ophiopogonin O/isomer
20	2.39	$C_{39}H_{61}O_{14}S$	75.35	4.96×10^{6}	801.3701	MS^2[801]: 655[M-H-Rha]$^-$, 637[M-H-Rha-H_2O]$^-$	ophiopogonin C'/isomer-sulfate
21	2.36	$C_{51}H_{81}O_{22}$	53.64	5.47×10^{5}	1045.5197	MS^2[1045]: 899[M-H-Rha]$^-$, 737[M-H-Rha-Glc]$^-$, 577[M-H-Rha-Glc-Glc-H_2O]$^-$	Rha(1→?)-Glc(1→?)pennogenin 3-*O*-*α*-L-Rha-(1→2)-*β*-D-Glc/isomer
22	2.35	$C_{49}H_{73}O_{15}S$	30.73	4.16×10^{5}	933.4659	MS^2[933]: 787[M-H-Rha]$^-$, 771[M-H-Rha-H_2O]$^-$, 625[M-H-Rha-Glc]$^-$, 463[M-H-Rha-2Glc]$^-$	Ophiopogonin D/isomer-sulfate
23	2.33	$C_{38}H_{59}O_{15}S$	69.13	6.84×10^{6}	787.3599	MS^2[787]: 713[M-H-64]$^-$, 641[M-H-Rha]$^-$, 623[M-H-Rha-H_2O]$^-$	14-hydroxy diosgenin 3-*O*-*β*-D-Xyl-(1→4)-*β*-D-Glc/isomer-sulfite
24	2.31	$C_{45}H_{71}O_{19}$	63.15	6.58×10^{6}	915.4591	MS^2[915]: 897[M-H-H_2O]$^-$, 769[M-H-Rha]$^-$, 589[M-H-Glc-H_2O]$^-$	(25R)-14*α*,17*α*-hydroxyspirost-5-en-3*β*-yl 3-*O*-*α*-L-rhamnpyranosyl-(1→2)-*β*-D-glucopyranosyl-(1→3)-*β*-D-glucopyranoside/isomer
25	2.24	$C_{56}H_{91}O_{28}$	31.86	7.03×10^{5}	1211.5653	MS^2[1211]: 1193[M-H-H_2O]$^-$, 1031[M-H-Glc-H_2O]$^-$	Ophiofurospiside C/isomer
26	2.21	$C_{50}H_{81}O_{25}S$	27.38	1.83×10^{6}	1113.4742	MS^2[1113]: 1095[M-H-H_2O]$^-$, 981[M-H-Xyl]$^-$, 951[M-H-Glc]$^-$, 933[M-H-Glc-H_2O]$^-$, 871[M-H-Glc-80]$^-$	ophiopogoside A/isomer-sulfate
27	2.20	$C_{51}H_{83}O_{26}S$	22.46	5.00×10^{5}	1143.4850	MS^2[1143]: 1125[M-H-H_2O]$^-$, 997[M-H-Rha]$^-$, 981[M-H-Glc]$^-$, 835[M-H-Glc-Rha]$^-$, 817[M-H-Glc-Rha-H_2O]$^-$, 801[M-H-2Glc-Rha-H_2O]$^-$, 753[M-H-Glc-64-Rha-H_2O]$^-$	Rha-(1→?) ophiogenin 3-*O*-*α*-L-Rha-(1→2)-*β*-*D*-Glc/isomer-sulfite
28	2.17	$C_{45}H_{73}O_{21}S$	25.0	7.14×10^{5}	981.4367	MS^2[981]: 835[M-H-Rha]$^-$, 819[[M-H-Glc]$^-$, 801[M-H-Glc-H_2O]$^-$, 673[M-H-Rha-Glc]$^-$, 655[M-H-Rha-Glc-H_2O]$^-$	protobioside/isomer-sulfate or ophiopojaponin B/isomer-sulfite
29	2.14	$C_{45}H_{73}O_{21}S$	16.62	9.60×10^{4}	981.4335	MS^2[981]: 835[M-H-Rha]$^-$, 819[M-H-Glc]$^-$, 801[M-H-Glc-H_2O]$^-$,	protobioside/isomer-sulfate or

						673[M-H-Glc-Rha]$^-$, 655[M-H-Glc-H_2O-Rha]$^-$	ophiopojaponin B/isomer-sulfite
30	1.99	$C_{51}H_{83}O_{28}S$	15.90	5.18×10^4	1175.4757	MS^2[1175]: 1093[M-H-64-H_2O]$^-$, 1029[M-H-Rha]$^-$, 1013[M-H-Glc or M-H-64-H_2O-80]$^-$, 947[M-H-64-H_2O-Rha]$^-$, 931[M-H-64-H_2O-Glc]$^-$, 785[M-H-64-H_2O-Glc-Rha]$^-$, 767[M-H-64-2H_2O-Glc-Rha]$^-$, 605[M-H-64-2H_2O-2Glc-Rha]$^-$	?-hydroxy Xyl (1→?) Rha (1→?) pennogenin 3-*O*-*α*-L-Rha-(1→2)-*β*-*D*-Glc/isomer-sulfite-sulfate
31	1.92	$C_{51}H_{83}O_{27}S$	31.07	1.92×10^6	1079.5242	MS^2[1079]: 1119[M-H-H_2O-42]$^-$, 933[M-H-Rha]$^-$, 917[M-H-Glc]$^-$, 771[M-H-Rha-Glc]$^-$, 753[M-H-Rha-Glc-H_2O]$^-$, 691[M-H-Rha-Glc-80]$^-$	Ac (1→?) protobioside/isomer-sulfate
32	1.86	$C_{51}H_{83}O_{26}S$	20.47	6.79×10^5	1143.4845	MS^2[1143]: 981[M-H-Glc]$^-$, 917[M-H-Glc-64]$^-$, 819[M-H-2Glc]$^-$, 801[M-H-2Glc-H_2O]$^-$, 771[M-H-Glc-64-Rha]$^-$, 609[M-H-2Glc-64-Rha]$^-$, 591[M-H-2Glc-64-Rha-H_2O]$^-$	Glc (1→?) ophiopojaponin B/isomer-sulfite
33	1.85	$C_{50}H_{81}O_{26}S$	26.64	2.98×10^6	1129.4928	MS^2[1129]: 1111[M-H-H_2O]$^-$, 983[M-H-Rha]$^-$, 967[M-H-Glc]$^-$, 949[M-H-Glc-H_2O]$^-$, 903[M-H-Rha-80]$^-$, 723[M-H-Rha-80-Glc-H_2O]$^-$, 591[M-H-Rha-80-Glc-H_2O-Xyl]$^-$, 429[M-H-Rha-80-2Glc-H_2O-Xyl]$^-$	Xyl (1→?) ophiopojaponin B/isomer-sulfate
34	1.85	$C_{51}H_{83}O_{25}S$	27.60	4.41×10^5	1127.4906	MS^2[1127]: 981[M-H-Rha]$^-$, 965[M-H-Glc]$^-$, 901[M-H-Glc-64]$^-$, 803[M-H-2Glc]$^-$, 785[M-H-2Glc-H_2O]$^-$	trigoneoside Iva/isomer-sulfite or Xyl (1→?) ophiopojaponin B/isomer-sulfite
35	1.78	$C_{51}H_{83}O_{26}S$	14.45	4.60×10^5	1143.4843	MS^2[1143]: 981[M-H-Glc]$^-$, 963[M-H-Glc-H_2O]$^-$, 917[M-H-Glc-64]$^-$, 771[M-H-Glc-64-Rha]$^-$, 753[M-H-Glc-64-Rha-H_2O]$^-$, 591[M-H-2Glc-64-Rha-H_2O]$^-$	Glc (1→?) ophiopojaponin B/isomer-sulfite
36	1.77	$C_{51}H_{83}O_{25}S$	24.36	5.68×10^4	1127.5088	MS^2[1127]: 981[M-H-Rha]$^-$, 965[M-H-Glc]$^-$, 901[M-H-Glc-64]$^-$, 803[M-H-2Glc]$^-$, 785[M-H-2Glc-H_2O]$^-$, 755[M-H-Glc-64-Rha]$^-$, 593[M-H-2Glc-64-Rha]$^-$, 413[M-H-3Glc-64-Rha-H_2O]$^-$	trigoneoside Iva/isomer-sulfite or Xyl-(1→?) ophiopojaponin B/isomer-sulfite
37	1.77	$C_{52}H_{83}O_{26}S$	31.47	1.20×10^6	1155.4867	MS^2[1155]: 1137[M-H-H_2O]$^-$, 1095[M-H-H_2O-42]$^-$, 1023[M-H-Xyl]$^-$, 993[M-H-Glc]$^-$, 849[M-H-2Xyl-42]$^-$	Ac (1→?) ophiopogoside A/isomer-sulfate
38	1.72	$C_{45}H_{73}O_{21}S$	41.22	1.51×10^6	981.4362	MS^2[981]: 835[M-H-Rha]$^-$, 819[M-H-Glc]$^-$, 801[M-H-Glc-H_2O]$^-$, 689[M-H-2Rha]$^-$, 609[M-H-2Rha-80]$^-$, 447[M-H-2Rha-80-Glc]$^-$,	protobioside/isomer-sulfate

						429[M-H-2Rha-80-Glc-H_2O]$^-$	
39	1.71	$C_{56}H_{91}O_{30}S$	22.25	1.96×10^6	1275.5287	MS^2[1275]: 1143[M-H-Xyl]$^-$, 1129[M-H-Xyl-H_2O]$^-$, 1113 [M-H-Glc]$^-$, 951[M-H-Glc]$^-$, 815[M-H-Glc-Rha]$^-$, 771[M-H-2Glc-H_2O]$^-$, 591[M-H-3Glc-2H_2O]$^-$	ophiopogonin F/G/isomer-sulfate
40	1.69	$C_{51}H_{83}O_{26}S$	21.97	5.24×10^6	1143.4880	MS^2[1143]: 997[M-H-Rha]$^-$, 981[M-H-Glc]$^-$, 817[M-H-Rha-Glc-H_2O]$^-$, 753[M-H-Rha-Glc-H_2O-64]$^-$	Rha-(1→?) ophiogenin 3-*O*-*α*-L-Rha-(1→2)-*β*-*D*-Glc/isomer-sulfite
41	1.56	$C_{51}H_{83}O_{28}S$	15.97	7.88×10^5	1175.4727	MS^2[1175]: 1093[M-H-64-H_2O]$^-$, 1013[M-H-Glc or M-H-64-H_2O-80]$^-$, 947[M-H-64-H_2O-Rha]$^-$, 931[M-H-64-H_2O-Glc]$^-$, 785[M-H-64-H_2O-Glc-Rha]$^-$, 767[M-H-64-2H_2O-Glc-Rha]$^-$, 605[M-H-64-2H_2O-2Glc-Rha]$^-$	?-hydroxy Xyl (1→?) Rha (1→?) pennogenin 3-*O*-*α*-L-Rha-(1→2)-*β*-*D*-Glc/isomer-sulfite-sulfate
42	1.56	$C_{52}H_{83}O_{25}S$	40.92	2.3×10^5	1139.4911	MS^2[1139]: 1097[M-H-42]$^-$, 1079[M-H-42-H_2O]$^-$, 993[M-H-Rha]$^-$, 977[M-H-Glc]$^-$, 965[M-H-42-Xyl]$^-$, 901[M-H-42-Xyl-80]$^-$, 833[M-H-42-2Xyl]$^-$, 755[M-H-42-Xyl-80-Rha]$^-$, 575[M-H-42-Xyl-80-Rha-Glc-H_2O]$^-$	Ac (1→?) ophiopogoside A/isomer-sulfIte
43	1.55	$C_{52}H_{83}O_{26}S$	39.96	5.09×10^5	1155.4850	MS^2[1155]: 1137[M-H-H_2O]$^-$, 1095[M-H-H_2O-42]$^-$, 1023[M-H-Xyl]$^-$, 993[M-H-Glc]$^-$, 849[M-H-2Xyl-42]$^-$	Ac (1→?) ophiopogoside A/isomer-sulfate
44	1.53	$C_{52}H_{83}O_{27}S$	30.14	5.04×10^5	1171.4795	MS^2[1171]: 1153[M-H-H_2O]$^-$, 1111[M-H-H_2O-42]$^-$, 1025[M-H-Rha]$^-$, 1009[M-H-Rha-H_2O]$^-$, 997[M-H-42-Xyl]$^-$, 991[M-H-Glc-H_2O]$^-$, 769[M-H-Glc-H_2O-Xyl]$^-$, 689[M-H-Glc-H_2O-Xyl-80]$^-$	Ac (1→?) Xyl (1→?) ophiopojaponin B/isomer-sulfate
45	1.53	$C_{39}H_{61}O_{14}S$	74.99	5.54×10^6	801.3694	MS^2[801]: 655[M-H-Rha]$^-$, 637[M-H-Rha-H_2O]$^-$	ophiopogonin C'/isomer-sulfate
46	1.51	$C_{45}H_{73}O_{21}S$	16.65	7.14×10^5	981.4360	MS^2[981]: 835[M-H-Rha]$^-$, 819[M-H-Glc]$^-$, 801[M-H-Glc-H_2O]$^-$, 771[M-H-Rha-64]$^-$, 673[M-H-Glc-Rha]$^-$, 655[M-H-Glc-Rha-H_2O]$^-$, 493[M-H-2Glc-Rha-H_2O]$^-$	ophiopojaponin B/isomer-sulfite
47	1.50	$C_{50}H_{81}O_{27}S$	19.39	4.83×10^5	1145.4669	MS^2[1145]:1127[M-H-H_2O]$^-$, 999[M-H-Rha]$^-$, 983[M-H-Glc]$^-$, 767[M-H-Rha-Xyl]$^-$, 605[M-H-Rha-Xyl-Glc]$^-$, 443[M-H-Rha-Xyl-2Glc]$^-$	?-hydroxy -trigonepside Iva/isomer-sulfate

Notes: _: characteristic product ions; Glc: glucopyranosyl; Xyl: xylopyranosyl; Rha:rhamnopyranosyl; Ac: acetyl; ?: could not ascertain the linking position.

Table S3. The detail information of in commercial raw materials.

Code No.	Name	Location
JSPACM-01-1	*Ophiopogon japonicus*	Chengdu city, Sichuan, China
JSPACM-01-2$^{\Delta}$	*Ophiopogon japonicus*	Chengdu city, Sichuan, China
JSPACM-02-1	*Ophiopogon japonicus*	Chengdu city, Sichuan, China
JSPACM-02-2$^{\Delta}$	*Ophiopogon japonicus*	Chengdu city, Sichuan, China
JSPACM-03-1	*Ophiopogon japonicus*	Chengdu city, Sichuan, China
JSPACM-03-2$^{\Delta}$	*Ophiopogon japonicus*	Chengdu city, Sichuan, China
JSPACM-04-1	*Ophiopogon japonicus*	Chengdu city, Sichuan, China
JSPACM-04-2$^{\Delta}$	*Ophiopogon japonicus*	Chengdu city, Sichuan, China
JSPACM-05-1	*Ophiopogon japonicus*	Chengdu city, Sichuan, China
JSPACM-05-2$^{\Delta}$	*Ophiopogon japonicus*	Chengdu city, Sichuan, China
JSPACM-06-1	*Ophiopogon japonicus*	Chengdu city, Sichuan, China
JSPACM-06-2$^{\Delta}$	*Ophiopogon japonicus*	Chengdu city, Sichuan, China
JSPACM-07-1	*Ophiopogon japonicus*	Chengdu city, Sichuan, China
JSPACM-07-2$^{\Delta}$	*Ophiopogon japonicus*	Chengdu city, Sichuan, China
JSPACM-08-1	*Ophiopogon japonicus*	Chengdu city, Sichuan, China
JSPACM-08-2$^{\Delta}$	*Ophiopogon japonicus*	Chengdu city, Sichuan, China
JSPACM-09-1	*Ophiopogon japonicus*	Chengdu city, Sichuan, China
JSPACM-09-2$^{\Delta}$	*Ophiopogon japonicus*	Chengdu city, Sichuan, China
JSPACM-10-1	*Ophiopogon japonicus*	Anguo city, Hebei, China
JSPACM-10-2$^{\Delta}$	*Ophiopogon japonicus*	Anguo city, Hebei, China
JSPACM-11-1	*Ophiopogon japonicus*	Bozhou city, Anhui, China
JSPACM-11-2$^{\Delta}$	*Ophiopogon japonicus*	Bozhou city, Anhui, China
JSPACM-12-1	*Ophiopogon japonicus*	Zhangshu city, Jiangxi, China
JSPACM-12-2$^{\Delta}$	*Ophiopogon japonicus*	Zhangshu city, Jiangxi, China
JSPACM-13-1	*Ophiopogon japonicus*	Hangzhou city, Zhejiang, China
JSPACM-13-2$^{\Delta}$	*Ophiopogon japonicus*	Hangzhou city, Zhejiang, China

Figure S1. The NIR spectra (4000-10000 cm-1) for 26 batches of collected samples

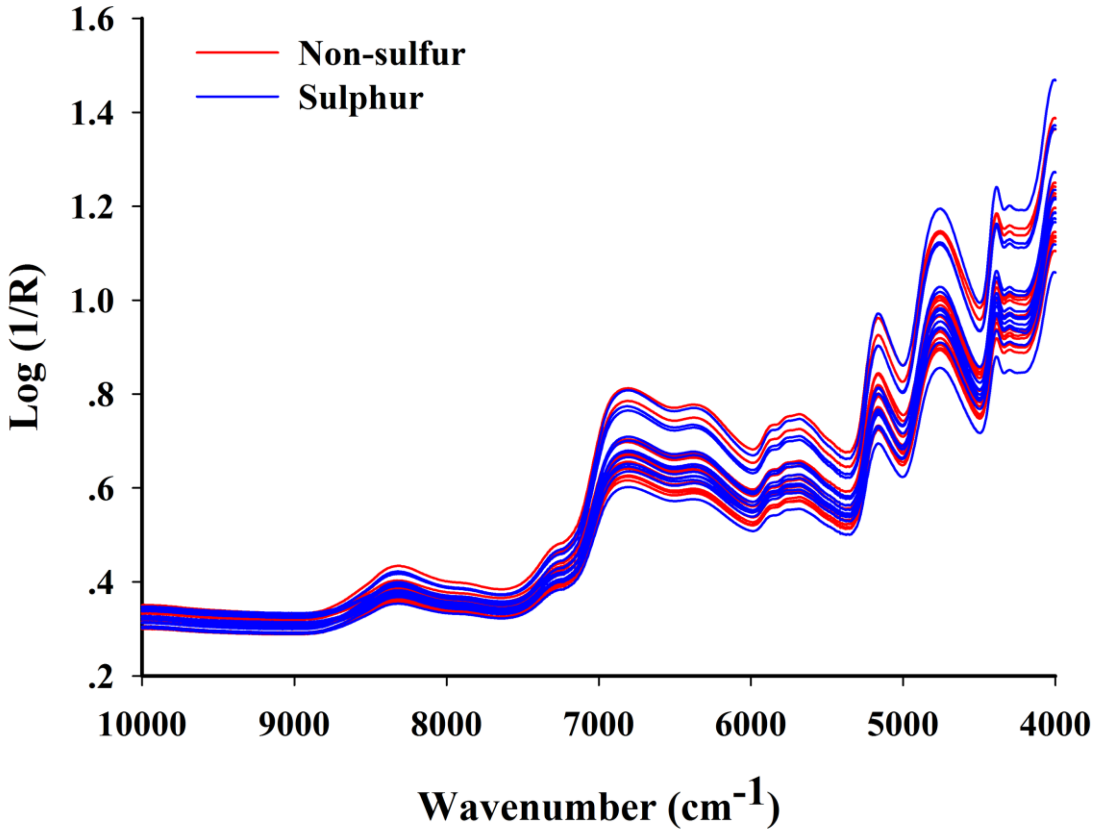

Figure S2. The discriminatory information in the different preprocessing methods for PLS-DA model.

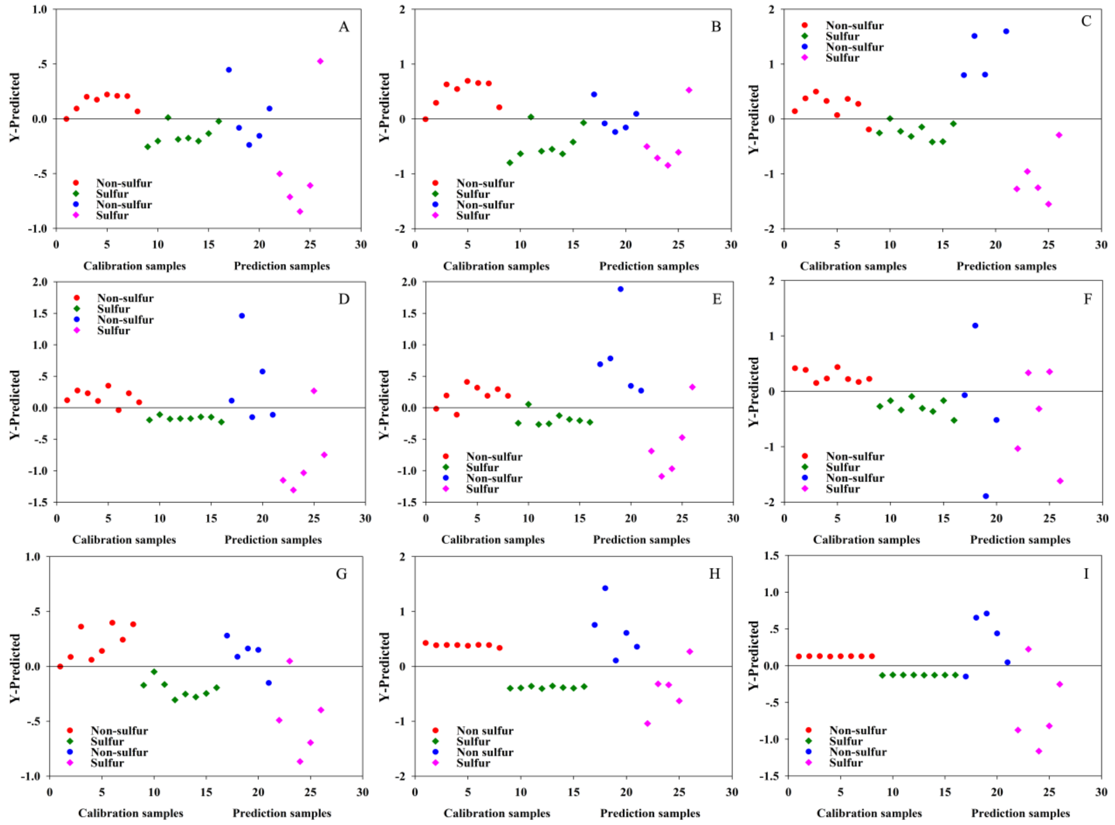

A multiplicative scatter correction (MSC), **B**: standard normal variate transformation (SNV), **C**: baseline, **D**: normalization, **E**: spectroscopic transformation (ST), **F**: wavelet denosing of spectra (WDS), **G**: Savitzky-Golay smoothing with 9 points SG(9) plus second-order derivatives, **H**: SG(11) plus first-order derivatives, and **I**: SG(11) plus second-order derivatives

Figure S3. HPLC spectra of JSPACM-05-1 and JSPACM-05-2 (A: ELSD; B: DAD at 296 nm).

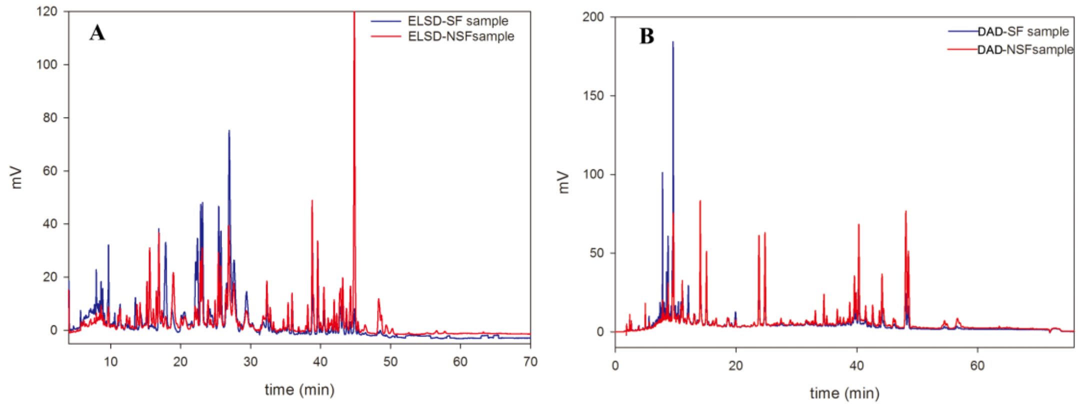

Figure S4. The PLS-DA (A) model of Ophiopogonis Radix using the HPLC-DAD-ELSD data; (B) The PLS-DA model was considered as valid significantly since the corresponding Q^2-intercept value is negative; (C) S-plot based on the HPLC-DAD-ELSD data.

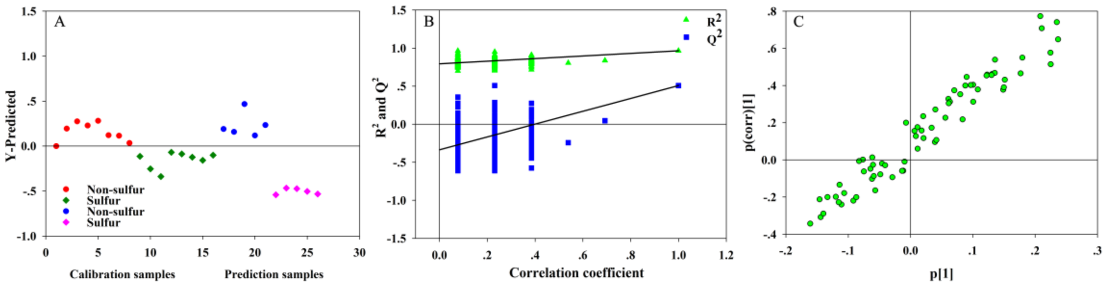

Figure S5 LC-MS spectra of JSPACM-05-1 and JSPACM-05-2.

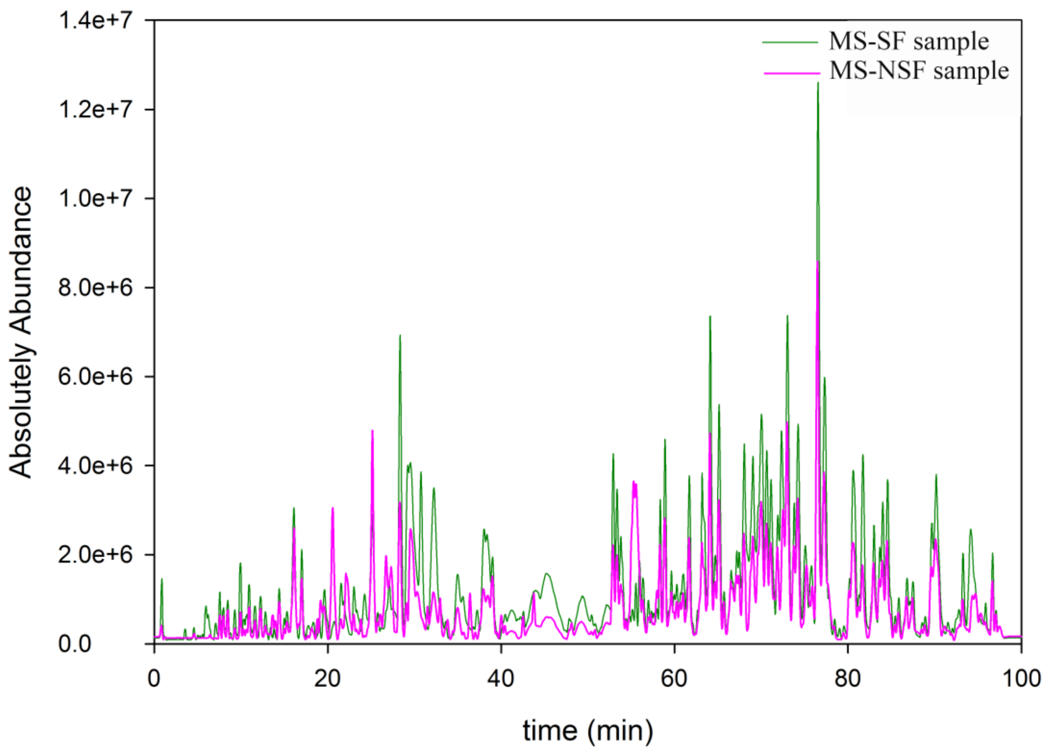

.

Figure S6. The contour plot of synchronous spectra of NSF (A) and SF (B) of Ophiopogonis Radix.

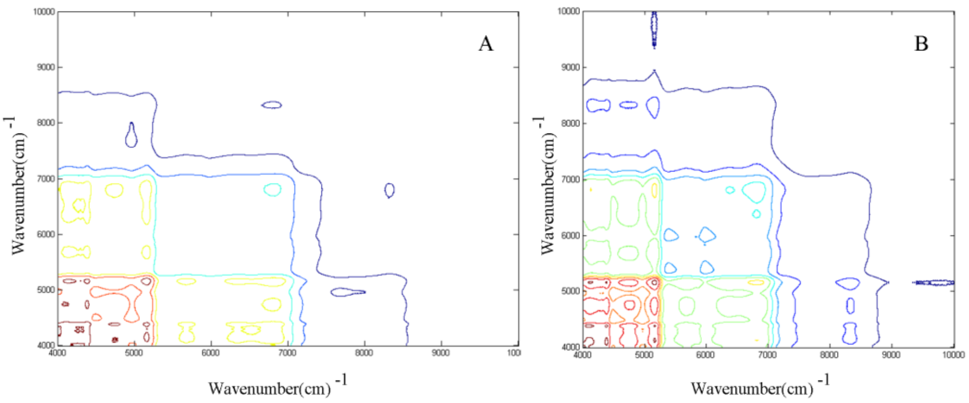

Figure S7. The contour plot of synchronous spectra of NSF (A) and SF (B) of Ophiopogonin D.

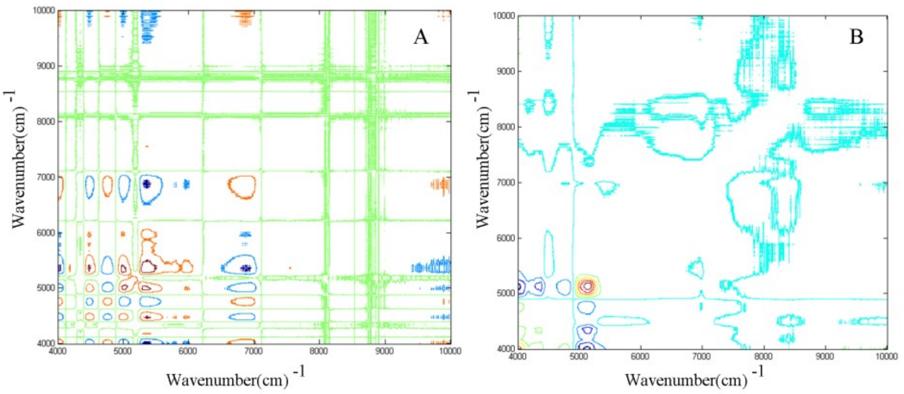

Figure S8 HRMS spectra of ophionponin D and its sulfate derivatives (A: ophiopogonin D; B: sulfate erivatives).

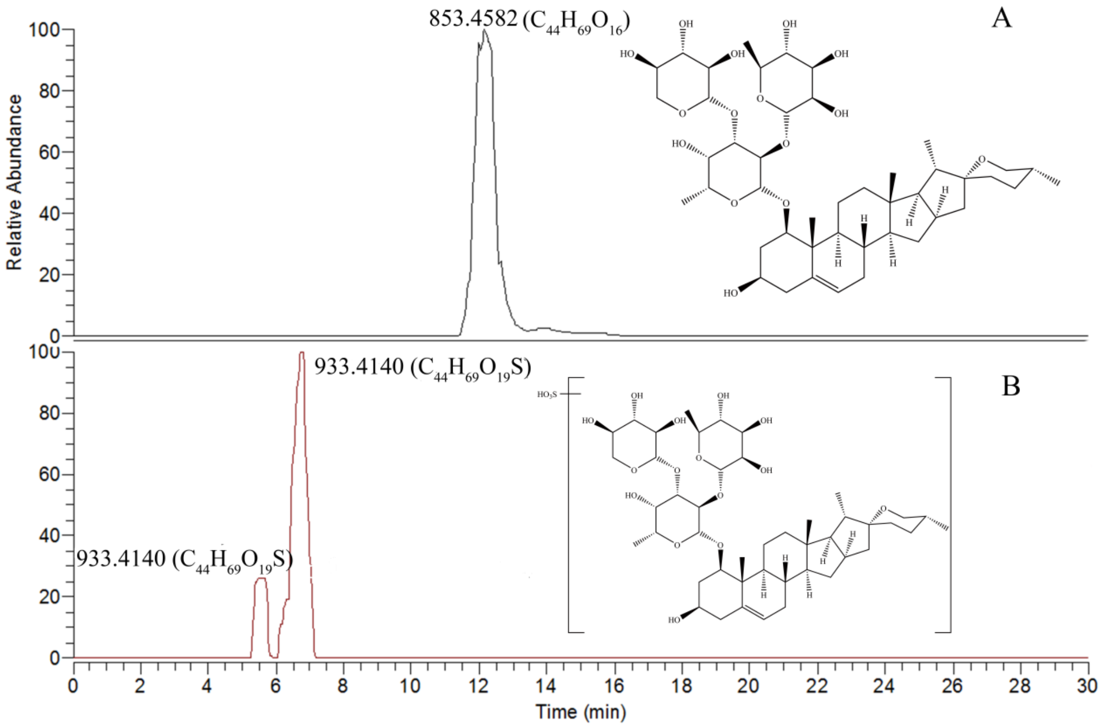

Supplement: Supplementary file 1 — Supplementary information [file 41598_2017_10313_MOESM1_ESM.pdf]
